# Extracellular Vesicles as Therapeutic Tools in Cardiovascular Diseases

**DOI:** 10.3389/fimmu.2014.00370

**Published:** 2014-08-04

**Authors:** Audrey Fleury, Maria Carmen Martinez, Soazig Le Lay

**Affiliations:** ^1^INSERM U1063 “Oxidative Stress and Metabolic Pathologies,” Institut de Biologie en Santé, Université d’Angers, Angers, France

**Keywords:** extracellular vesicle, microparticles, microvesicles, exosomes, cardiovascular diseases, angiogenesis, sonic hedgehog, therapeutical tools

## Abstract

Extracellular vesicles (EVs), including microvesicles (MVs) and exosomes, are small vesicles secreted from a wide variety of cells. Whereas MVs are particles released by the outward budding of the plasma membrane, exosomes are derived from endocytic compartments. Secretion of EVs can be enhanced by specific stimuli, and increased plasma circulating levels of EVs have been correlated with pathophysiological situations. MVs, already present in the blood of healthy individuals, are considerably elevated in several cardiovascular diseases associated with inflammation, suggesting that they can mediate deleterious effects such as endothelial dysfunction or thrombosis. Nonetheless, very recent studies also demonstrate that MVs may act as biological information vectors transferring proteins or genetic material to maintain cell homeostasis, favor cell repair, or even promote angiogenesis. Additionally, exosomes have also been shown to have pro-angiogenic and cardio-protective properties. These beneficial effects, therefore, reveal the potential therapeutical use of EVs in the field of cardiovascular medicine and regenerative therapy. In this review, we will provide an update of cellular processes modulated by EVs of specific interest in the treatment of cardiovascular pathologies. A special focus will be made on the morphogen sonic hedgehog (Shh) associated with EVs (EVs^Shh+^), which have been shown to mediate many pro-angiogenic effects. In addition to offer a potential source of cardiovascular markers, therapeutical potential of EVs reveal exciting opportunities to deliver specific agents by non-immunogenic means to cardiovascular system.

## Introduction

Extracellular vesicles (EVs) comprising exosomes (<100 nm of size), microparticles, and apoptotic bodies (100–1200 nm in size) are physiologically released by almost all cell types and represent endogenous cargos that are able to participate in cell-to-cell communication. The composition of EVs determines the type of message, which they can convey. Indeed, EVs are rich in proteins, mRNAs, and miRNAs as well as lipids, which they can transfer to target cells by ligand/receptor interaction, fusion, and/or internalization. Manipulation of cells generating EVs by pharmacological treatment or transfection allow to produce EVs rich in specific components that can affect selectively the functions of target cells. In this article, we will review the current knowledge of the function and impact of EVs used as therapeutic tools against cardiovascular diseases.

## EVs: Potential Delivery Tools/Vectors in the Cardiovascular System

In addition to their different size, the criteria that are used to distinguish between the various types of EVs are based on the cellular compartment that they originate from and on their composition (1). While exosomes are formed from the multivesicular bodies, both microparticles and apoptotic bodies come from plasma membrane. Obviously, composition of EVs depends on the type of (i) cells that they are from and (ii) stimulation that generates their release. Thereby, specific antigens carried by EVs allow the identification of donor/producer cell, whereas use of different agents to stimulate EV generation allows achieving EVs with different potential effects.

Extracellular vesicles have been isolated from the main fluids of the organism and from several solid tissues (tumors, atheroma plaque, skeletal muscle…). However, it is difficult to establish the exact nature of EVs from solid tissues, as the recovered materials can also comprise intracellular vesicles released during the tissue dissociation process (2). In addition to their physiological role during development and homeostasis, some pathophysiological situations will enhance release of EVs and affect composition of EVs or both. Thus, during cardiovascular diseases, both elevated levels and different composition of EVs have been described suggesting that EVs may represent potential novel targets for therapeutic intervention (3, 4). Of note, circulating endothelial EVs were associated with the presence of cardiometabolic risk factors in a community cohort underscoring the potential influence of high-risk metabolic profiles on endothelium activation or injury (5). Given the fact that elevated EV levels often correlate with the severity of cardiovascular diseases, one strategy would consist in reducing circulating EVs to normal levels in order to prevent associated deleterious signaling. In this perspective, many ongoing studies are designed to evaluate changes in circulating EV levels in response to pharmacological treatment used [for review, see Ref. (6)]. However, such strategies do not allow distinguishing between effects of drugs on EV generation and/or EV clearance. An alternative to modulate EV production would be either to act on processes regulating their formation and/or release or to inhibit their interaction with target cells through specific targeting of their components (receptors, ligands, lipids…) (7). Notably, inhibition of ceramide formation by the use of amiloride drug reduces significantly mouse and human tumor cell growth by blocking secretion of heat shock protein-72 (HSP 72) EV-associated protein (8). While no studies using such strategies have been yet described in cardiovascular field, a limiting step is also linked to the lack of specificity of targeting mechanisms regulating EV formation and release, which may produce many unwanted effects.

Alternatively, EVs constitute promising therapeutic delivery tools in cardiovascular diseases. Their general capacity to act as bioactive cargoes, particularly through their ability to carry secretory molecules such as cytokines, chemokines, or growth factors as well as exogenous nucleic acids, highlight them as attractive vehicles (9). In addition, EVs are able to use the vasculature to signal at considerable distance from their donor cell origin. In this respect, central nervous system-derived EVs could enter the bloodstream and communicate with endothelial cells in the peripheral circulation and with cells involved in immune surveillance (10).

**Figure 1:**
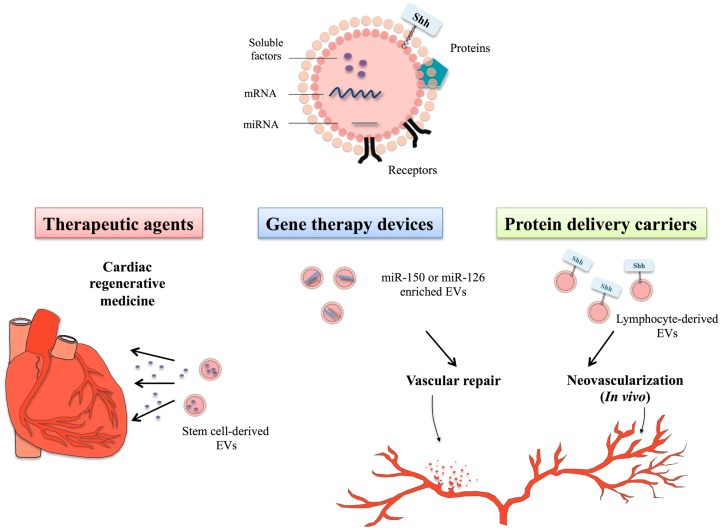
**Therapeutical potential of extracellular vesicles in the context of cardiovascular disorders**. Extracellular vesicles (EVs) constitute bioactive cargoes, particularly through their ability to carry proteins, receptors but also genetic material including microRNA (miRNA) or mRNA. Since EVs participate actively in the modulation of many physiological processes, they can be used as therapeutic agents in cardiac regenerative medicine. Improvement of cardiac function depends on the EV-transfer of specific factors, such as soluble proteins, growth factors, lipids, or genetic material. Use of stem cell-derived EVs in cardiac regenerative therapies, therefore, allows delivering specific signals by non-immunogenic means to heart. As EVs naturally contain genetic material, cell sources can be genetically manipulated to produce EVs specifically loaded with miRNA of choice, such as miR-150 or miR-126, described as important regulators of angiogenesis or vascular integrity. Treatment of endothelial cells or vascular tissues with these miRNA-enriched EVs has demonstrated their protective and regenerative properties on cardiovascular system. Specific culture system might also allow to specifically targeting molecules of interest to EVs, like the morphogen Shh that is an important regulator of injury-induced angiogenesis and neovascularization. Use of EVs^Shh+^ even appears in this context more powerful than classical recombinant Shh protein, likely linked to its localization within natural EV membranous environment.

Although largely identified as deleterious carriers, EVs might also have beneficial effects and deliver protective messages to preserve endothelial function and/or vascular integrity. Indeed, circulating EVs from sepsis patients are able to restore vascular hyporeactivity in vessels treated by lipopolysaccharide (11). In agreement, high circulating levels of EVs correlate with survival in sepsis patients (12). Besides this EV-mediated adaptative/protective response, EVs have demonstrated regenerative capacities, mainly when they derived from progenitor or mesenchymal stem cells (MSC), therefore, opening perspectives to use their innate properties in regenerative medicine (Figure 1).

## Therapeutic Use of MSC-Derived EVs in Cardiac Regenerative Medicine

Cardiac regenerative therapy, based on intramyocardial injection of MSCs, has been in the last few years one of the most promising approaches to both prevent cardiac damage and allow cardiac repair. However, experimental or clinical studies demonstrate insufficient cardiomyocyte or vascular cell differentiation to account for improvements of cardiac function (13). This led to formulate the paracrine-hypothesis according to which soluble factors derived from MSCs would be responsible for beneficial outcomes (14, 15). First evidences establishing that cellular secretions are cardio-protective resulted from administration of MSC-derived conditioned medium to rodents and pigs, which results in reduced infarct size and led to improved heart function (16–18). Also, exosomal fraction contained in MSC-conditioned medium mediates preservation of cardiac function after ischemia/reperfusion injury (19). These protective effects likely result from decreased oxidative stress and activation of PI3K/Akt pathway, which enhance myocardial viability and prevent adverse cardiac remodeling (20). Of note, EVs derived from bone marrow CD34^+^ stem cells or endothelial progenitor cells (EPCs) have been shown to promote angiogenesis (21, 22).

Of great interest is the recently reported potential of EVs, particularly exosomes, derived from cardiac progenitor cells (CPC). Indeed, CPC-derived EVs have been shown to stimulate migration of endothelial cells (23) and to protect ischemic myocardium from acute ischemia/reperfusion injury (24). This would, therefore, open new perspectives to directly used EVs derived from cells present in the heart itself. Accordingly, electron microscopic imaging of CPCs and adult mouse hearts has revealed the release of vesicles, which would have the morphological characteristics of EVs (25).

Altogether, the use of stem cell-derived EVs instead of stem cells engraftment might open new perspectives for cardiac regenerative therapies. This would first resolve safety concerns related to uncontrolled dissemination of transplanted cells or aberrant stem cell differentiation. In addition, immunosuppressive effects reported for certain EVs could favor efficient cardiac tissue regeneration (26). Moreover, since exosomes could be easily stored and keep their biological properties over an extended storage period, they can overcome many of the limitations linked to the use of viable cells in regenerative medicine. However, further investigations would be needed to optimize their characterization, quality, and purification in order to accurately control their production in the perspective of clinical uses (27). In particular, pro or anti-tumor effects reported for MSC-derived EVs would need to be fully understood and highly regulated (28, 29).

## EVs as Gene-Therapy Devices

As described above, EV composition and cargoes transported (proteins, genetic material, lipids) vary with cell origin. In addition to use them as delivery vector for specific proteins, their ability to transport and deliver genetic material to non-adjacent cells have highlighted their potential as new delivery vectors in gene-therapy (see Figure 1).

Extracellular vesicles derived from stem cells are reported as carriers for nucleic acids, which by horizontal transfer of mRNAs are able to reprogram hematopoietic progenitors (30). Subsequently, EPCs are also shown to activate angiogenesis in endothelial cells by shuttling specific mRNA-derived EVs associated with the phosphoinositide 3-kinase (PI3K)/AKT signaling pathway, therefore, inducing angiogenic responses (31).

Further investigations have demonstrated that delivery of mRNAs was not restricted to MVs but could be extended to exosomes derived from mast cells (32) and could be translated once after entering another cell (32–34). Furthermore, detailed RNA analysis of total RNAs derived from EVs from various cell types also revealed the presence of small RNAs, including microRNAs (miRNA) (32, 35, 36). Transfer of miRNAs contained in exosomes derived from dendritic cells operates via fusion of cells, leading to the release of the exosomes content into the dendritic cell cytosol (37).

Numerous clinical studies have revealed that cardiovascular diseases correspond with specific signature patterns of miRNA expression (38, 39). Circulating EVs represent transport vehicles for large numbers of specific miRNAs involved in fundamental cellular processes related to cardiovascular disorders (40). Importantly, the comparison of ratio of miRNA expression in circulating EVs to that in EV-free plasma reveals the majority of circulating miRNAs were present in EVs (41). Since miRNA profile in EVs is significantly different from their maternal cells, an active mechanism of selective “packaging” from cells into EVs likely occurs. Of note, molecular packaging of miRNA-loaded exosomes is controlled through the recognition of sequence motifs present in miRNAs by sumoylated protein hnRNPA2B1 (heterogeneous nuclear ribonucleoprotein A2B1) (42). Moreover, this compartmentalization may be regulated by external stimuli as illustrated by hypoxia, which can modulate immunomodulatory and regenerative properties of MSC-derived EVs (16) but also enhanced expression of pro-angiogenic miRNAs in EPC-derived EVs (43).

MiRNAs contained in EVs might efficiently regulate target genes in recipient cells as illustrated with monocyte miRNA-150, selectively packaged into EVs following treatments of human monocyte/macrophage THP1 cells with inflammatory factors (41). By the use of THP-1-derived EVs expressing a mimic miR-150, the authors showed elevated miR-150 levels in *in vitro* EV-treated endothelial cells as well as in mouse blood vessels following EV intravenous injection. Consequently, protein level of c-Myb, a gene targeted by miR-150, is effectively reduced resulting in enhanced endothelial cell migration. This monocyte/macrophage EVs delivery system has been further optimized by transfecting donor cells with a chemically modified miRNA (miR-143), which revealed higher stability (44).

MiR-126, an important regulator of angiogenesis and vascular integrity (45, 46) is enriched in different types of EVs, such as apoptotic bodies derived from endothelial cells (47), endothelial microparticles (48), or even EPCs (49). In agreement with the striking evidence describing the role of miR-126 in the regulation of vascular integrity on the molecular level, treatment with these different types of miR-126-enriched EVs all promote vascular regeneration. Indeed, systemic treatment of hypercholesterolemic ApoE^-/-^ mice with miR-126-enriched EVs limited atherosclerosis and increased Sca1^+^ cell incorporation into aortic plaques (47) whereas miR-126-associated EVs promote reendothelialization *in vivo* (48). Alternatively, both angiogenic miR-126 and miR-296 participate in the improvement of neovascularization observed in a model of hindlimb ischemia induced in SCID mice following treatment with EPC-derived EVs (49). Of particular interest, miRNA profiling in patients with coronary artery disease has shown significantly reduced levels of miR-126 in comparison with healthy controls (50) or even total loss circulating miR-126 in patients with diabetes mellitus (39). Therefore, development of EV-based miR-126 therapeutic strategy might be promising in the context of metabolic and cardiovascular diseases.

Our group has developed one type of EVs carrying the morphogen Shh that reveals protective properties on cardiovascular cells. The next part of this review is focused on the description of the effects of these EVs.

## EVs Harboring Shh as Vascular and Cardio-Protective Delivery Tools

### Importance of Shh signaling in vascular function

Shh is a morphogen that belongs to the conserved hedgehog (Hh) protein family. Hedgehog signaling is essential in vertebrates for embryo patterning, as it participates in central nervous system development, lateral asymmetry, and anterior–posterior limb axis (51). In adult tissues, Shh signaling has been implicated in cell proliferation, differentiation, and survival in a wide variety of target tissues/organs [reviewed in detail in Ref. (52)]. Hh proteins are covalently linked to cholesterol at its carboxyl terminus (53) and palmitoylated at its amino terminus (54). Both lipid modifications influence activities of Shh (55, 56) and long-range spread of Shh signaling (57, 58). Diffusion of dual-lipid modified Shh proteins apparently occurs via secretion as mono or large soluble multimers, further transported via lipoproteins (59) or in EVs (60–62). A major component of the Hh pathway includes the 12 transmembrane receptor PTC (Patched) that constitutively represses Hh signaling. Binding of Shh to PTC inhibits the repression of Smoothened (SMO), which functions as G protein-coupled receptor (GPCR). This derepression leads to the activation of zinc-fingers transcriptions factors Gli, which translocate to the nucleus where they act as activators or repressors of target gene transcription. Besides this classical-Shh signaling pathway, referred as “canonical pathway,” other non-canonical pathways that do not clearly act via the Shh-Smo-Gli axis have been reported, which regulate many cellular processes including cell energy metabolism (63, 64).

**Figure 2:**
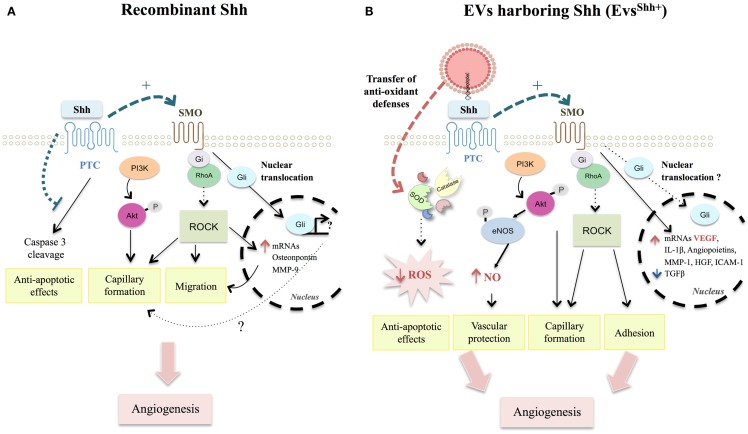
**Comparison between recombinant Shh- and EVs^Shh+^-activated pathways modulating angiogenesis and apoptosis in endothelial cells**. **(A)** Recombinant Shh protein was shown to promote migration and capillary formation through SMO-Gi coupled and PI3K activation, triggering ROCK pathway activation, and downstream targets (MMP-9 and osteopontin). Such activation illustrates the importance of non-canonical pathways in Shh-induced angiogenesis processes. Despite Shh induction of Gli nuclear translocation in endothelial cells, role of canonical signaling on vascular function remains elusive. Finally, activation of Hh signaling counteracts non-canonical PTC-induced caspase-3 cleavage and protects endothelial cells against apoptosis. **(B)** EVs^Shh+^ were demonstrated to use similar pathways than those described for recombinant Shh protein. They additionally induce functional and mature blood vessel formation by enhancing mRNA expression of several pro-angiogenic factors, especially VEGF and decreasing mRNA of anti-angiogenic TGFβ. EVs^Shh+^ also exert vasculoprotective effects by inducing anti-oxidants defenses, particularly through direct transfer of antioxidant enzymes (SOD, catalase) concurring to decrease ROS intracellular levels. SMO, smoothened; PI3K, phosphoinositide-3-kinase; ROCK, Rho-associated protein kinase; MMP-9, matrix metalloproteinase-9; Hh, Hedgehog; PTC, Patched; VEGF, vascular endothelial growth factor; TGFβ, transforming growth factor β; SOD, superoxide dismutase; ROS, reactive oxygen species.

Shh signaling is crucial in new vessel formation during embryogenesis and organogenesis. Its role in angiogenesis and neovascularization has been previously reviewed in details (65). Hh signaling components are present in adult cardiovascular tissues and can be activated *in vivo* (66). In adult tissue, activation of Shh signaling, via recombinant Shh or gene-therapy transfer, triggers cellular responses in a wide variety of vascular cells (endothelial, EPCs, smooth muscle cells, fibroblasts) promoting angiogenesis and protecting against ischemic injuries (66–68). These Shh effects on cardiovascular system seem to operate via complex pathways, involving Gli-dependent and independent pathways (Figure 2A).

Endothelial cells were initially considered not to respond to recombinant Shh directly, but rather to rely on adjacent mesenchymal cells thought to translate the presence of Hh ligand in signaling molecules that act on the endothelium (66). Particularly, activation of Shh signaling in intersticial mesenchymal cells enhances PTC, VEGF (Vascular Endothelial Growth Factor), and angiopoietin-1 and -2 protein expressions (66). Further studies demonstrate that endothelial cells do respond to Hh inducing pro-angiogenic responses (69, 70). PI3K/Akt and SMO dependencies have been demonstrated to be essential for Shh signaling (71, 72). In agreement, Shh acutely stimulates fibroblast migration and endothelial cell tubulogenesis through Rho GTPases Rac1 and RhoA activation depending on SMO-G(i)-coupling and PI3K, but independently of Gli transcription factors (69, 73). Similarly, Shh promotes capillary morphogenesis and endothelial cell migration via a RhoA/ROCK dependent pathway, ultimately leading to increased expression of metalloproteinase 9 (MMP-9) or osteopontin downstream targets (74). Despite Gli nucleus translocation following Shh treatment of endothelial cells, role of these transcription factors in Shh-angiogenesis responses is not fully understood (71). Controversy regarding Shh signaling is also present *in vivo* since Shh-induced angiogenesis is not affected in Gli1 null-mice (74), whereas capillaries morphogenesis is inhibited by the presence of Gli1 inhibitor GANT61 (70). Impaired angiogenesis in ischemic tissue of middle-aged or old mice is associated with a decrease in Gli1 transcription factor mRNA expression (68, 75). These results reflect the complex interplay between canonical and non-canonical Shh pathways, or at least that of the Gli family of transcription factors, in the regulation of tubulogenesis and angiogenesis processes.

### EVs harboring Shh as promising therapeutical devices for correcting angiogenic defects

Shh beneficial effects in angiogenesis reveal therapeutic potential for stimulating neovascularization in disease states associated with impaired angiogenesis. This has led our group to engineer EVs specifically enriched in this morphogen and derived from activated T-lymphocytic cells. EVs^Shh+^ are specifically retrieved from conditioned medium when lymphocytes are treated with a proactive stimulation, phytohemagglutin, and phorbol ester, followed by a pro-apoptotic treatment with actinomycin D (62). These EVs^Shh+^ are functionally active and induce differentiation of megakaryocytes. EVs^Shh+^ downstream effects likely depend on SMO activation, probably acting through direct Shh-PTC interaction (see Figure 2B). *In vitro* capillary formation by umbilical vein endothelial cells is both dependent on EVs^Shh+^ concentration and inhibited in presence of cyclopamine, which stabilizes SMO in an inactive form (76). However, whether EVs^Shh+^ induce activation of Gli1 remain to be determined. *In vitro*, EVs^Shh+^ increase mRNA levels of several pro-angiogenic factors, such as VEGF, angiopoietins, hepatocyte growth factor (HGF), interleukin-1β, MMP-1 and decrease anti-angiogenic factors such as transforming growth factor β (TGF β) (76). EVs^Shh+^ also increase cell adhesion and induce formation of stress fibers of *in vitro* endothelial cells in a SMO and Rho kinase pathways-dependent manner (76), likely signaling through the same pathway as described for recombinant Shh (69).

Angiogenic properties of EVs^Shh+^ have been illustrated following their *in vivo* intravenous injection in mouse models (77). *In vivo* injection of EVs^Shh+^ in mice is able to improve endothelial function by increasing nitric oxide (NO) release and reversing endothelial dysfunction after ischemia/reperfusion (78). Indeed, EVs^Shh+^ trigger changes in the expression and phosphorylation of enzymes related to NO pathway, which seem to be dependent of the activation of kinases such as PI3K and ERK. Furthermore, 21 day-treatment of a model of mouse hind limb ischemia with EVs^Shh+^ increase eNOS activation both in aorta and muscle as well as several pro-angiogenic factors (77). Taken together, lymphocytic-derived EVs^Shh+^ are able to induce functional and mature blood vessel formation by reciprocally regulating the pro- and anti-angiogenic factors.

Apart from directly stimulating angiogenesis processes, EVs^Shh+^ reveal anti-apoptotic properties notably by decreasing reactive oxygen species (ROS) production (78). Mechanistically, EVs^Shh+^ exert their vasculoprotective effects by promoting internalization and induction of antioxidant messages to the endothelial monolayer. Indeed, EVs^Shh+^ prevent apoptosis in endothelial cells induced by actinomycin D by transferring functional antioxidant enzymes [superoxide dismutase (SOD) isoforms and catalase] and by inducing cellular Mn-SOD protein expression (79). Anti-apoptotic effects have also been reported following Hh ligand treatment of endothelial cells, but rather relying on the inhibition of the pro-apoptotic effect of PTC, normally leading to caspase-3 cleavage (69).

Therefore, EVs^Shh+^ may represent a powerful tool in diseases associated with failed angiogenesis, associated with their pro-angiogenic and anti-apoptotic properties. In addition to observed differences in regulated pathways, structural features of Shh associated with EVs might be of particular importance. EVs provide a lipidic environment analogous to cell membrane, which could influence Shh downstream signaling considering the importance of these lipid-adducts in Shh activity (57, 58). Finally, EV-delivery of Shh protein seems to represent a major mechanism in the observed preservation of cardiac function in stem cell-based therapies. Indeed, CD34^+^ cells genetically modified to express Shh [CD34^+^(Shh)] offer a better protection against ventricular dilation and cardiac functional decline than CD34^+^ cells alone when injected into mice after acute myocardial infarction (80). *In vitro* analysis of exosomes derived from CD34^+^(Shh) further revealed the functional transfer and activation of Shh signaling in recipient cells, which might explain the observed preservation of cardiac function in mice treated with CD34^+^ (Shh) EVs.

Thus, EVs harboring Shh represent an exciting tool for therapy regarding angiogenesis defects related diseases and cardioprotection.

### Toward clinical translation

Extracellular vesicles might be further used as delivery devices for any molecule of interest. In particular, using naturally occurring secreted vesicles might allow overcoming toxicity or immunogenicity associated with other developed carrying agents like liposomes or nanoparticles. Moreover, this new drug-delivery system might be combined with other existing therapeutic strategies to optimize drug-delivery. Indeed, a recent report demonstrates that adeno-associated viruses (AAVs) are more efficient when encapsulated in EVs (termed vexosomes) than free AAVs for the delivery of cargo into recipient cells. Such a unique entity offers a promising strategy to improve gene delivery (81). Alternatively, tumor cells incubated with chemotherapeutic drugs are able to package these drugs into EVs, which can be collected and used to effectively kill tumor cells in murine tumor models without typical side effects (82).

Furthermore, the use of biological material as carriers might also allow getting molecules in their native conformation, due to natural interaction or folding with membranous environment. Different strategies might be used to specifically target molecules in EVs. For example, addition of different types of membrane anchors can target highly oligomeric cytoplasmic proteins to sites of vesicle secretion and EVs (83). Another important issue is the biodistribution of EVs. Indeed, EVs are circulating through many fluids, and have been especially recovered from blood or lymph, therefore allowing their large dissemination in the organism. Such circulating properties are of particular interest in the treatment of cardiovascular alterations since the entire vascular network would be exposed to EVs. Since dissemination or accumulation of these biological vectors may also be hindered, one could need to specifically target cargo delivery to enhance EV properties. Elegant studies using engineering donor cells expressing peptide targeting-ligand subsequently recovered in EVs produced by cells have respectively allowed to specifically target neurons (84) and breast cancer cells (85).

Despite interesting perspectives for cardiovascular treatment, EV-based therapies still need more investigations to translate into clinical studies. Lessons from the ongoing exosome-based preliminary clinical trials in the field of cancer would certainly boost such therapeutic perspectives. Particularly, one challenge would be to control the fragile balance between the harmful effects and beneficial ones reported for EVs in the context of cardiovascular diseases. However, cardiovascular field would undoubtedly benefit from optimized developed methods for large-scale production of clinical grade EVs derived from MSC (27) or dendritic cells (86).

By inhibiting their deleterious effects, by taking advantage of their regenerative properties or by developing EV-based drug-delivery tools, targeting EVs reveal all the exciting opportunities to treat cardiovascular alterations by non-immunogenic means. In particular, EVs carrying Shh^+^ represent interesting tools to treat diseases related to failed NO pathway and impaired angiogenesis.

## Conflict of Interest Statement

The authors declare that the research was conducted in the absence of any commercial or financial relationships that could be construed as a potential conflict of interest.
